# Vibration-Assisted Welding of 42CrMo4 Steel: Optimizing Parameters for Improved Properties and Weldability

**DOI:** 10.3390/ma17112708

**Published:** 2024-06-03

**Authors:** Mihai Alexandru Luca, Ionut Claudiu Roata, Cătălin Croitoru, Alina Luciana Todi-Eftimie

**Affiliations:** Department of Materials Engineering and Welding, Faculty of Materials Science and Engineering, Transylvania University of Brasov, B-dul Eroilor 29, 500036 Brasov, Romania; luca.mihai@unitbv.ro (M.A.L.); ionut.roata@unitbv.ro (I.C.R.); c.croitoru@unitbv.ro (C.C.)

**Keywords:** vibration-assisted welding, VAW, unconventional technology, weldability, vibration, stress relief, mechanical vibration, decreased internal friction

## Abstract

This study advances the vibration-assisted welding (VAW) technique for joining medium-carbon, low-alloy steels, which are typically challenging to weld. Traditional welding methods suggest low linear energy and mandatory pre- and post-heating due to these steels’ poor weldability. However, VAW employs a vibrating table to maintain part vibration throughout the automatic MIG/MAG welding process. This study tested the VAW technique on 42CrMo4 steel samples, achieving satisfactory weld quality without the need for pre- and post-heating treatments. This research revealed that while vibration frequencies between 550 Hz and 9.5 kHz minimally affect the appearance of the weld joint, the oscillation acceleration has a significant impact. The acceleration along the weld axis (a_x_), combined with the welding speed and vibration frequency, affects the weld surface’s appearance, particularly its scaly texture and size. Lateral acceleration (a_y_) alters the seam width, whereas vertical acceleration (a_z_) affects penetration depth at the root. Notably, if the effective acceleration (a_ef_) surpasses 40 m/s^2^, there is a risk of molten metal expulsion from the weld pool or piercing at the joint’s base. The quality of the joints was assessed through macroscopic and microscopic structural analyses, micro-hardness tests in the weld zone, and bending trials. The mechanical properties of the VAW samples were found to be acceptable, with hardness slightly exceeding that of the samples subjected to pre- and post-heating. Moreover, the VAW process significantly reduced energy consumption and operational time. The employed vibration system, with a power rating of 100 W, operates for just a few minutes, resulting in substantially lower energy usage compared to the traditional pre- and post-heating method, which typically requires a 5 kW electric furnace.

## 1. Introduction

Welding operations are fundamental in joining materials, particularly metals, by applying heat to the joint area, resulting in localized melting. However, this process induces uneven thermal stresses due to the expansion and contraction of the heated material. Initially, compressive stresses arise from the material’s expansion near the electric arc, leading to melting. As the metal cools, tensile stresses develop, potentially causing localized plastic deformations and permanent distortions in the welded components. Excessive stresses during welding and subsequent cooling can lead to cracks, particularly in the heat-affected zone (HAZ), if they surpass the tensile strength at a given temperature.

Cracks in the HAZ are often attributed to the base material’s chemical composition, which affects its physical and mechanical properties. With an increase in carbon content and alloying elements, steel’s deformability diminishes, and the likelihood of cracking escalates. To mitigate internal stresses and avert cracking, post-weld heat treatment (PWHT) is traditionally employed.

The inclusion of preheating equipment in the technological flow, as well as the welding of hot (preheated) materials and subsequent heat treatment, are costly operations that significantly hinder the technological flow and productivity, having a strong impact on the price of finished products. Therefore, the interest of researchers in finding an alternative technology to improve the weldability of steels with such a deficit is justified. The development of the VAW process began tentatively in the 2010s and has intensified in the last 5 years due to encouraging results. Thus, recent articles and reviews on this topic can be consulted in prestigious journals (with a very high number of citations), which indicates the academic interest in this direction [1,2,3].

This paper discusses experimental findings from welding tests on 42CrMo4 steel. This type of steel is known for its low weldability, necessitating specific welding conditions. The recommended MIG/MAG welding process involves a low linear energy regime and the absence of high internal stresses in the material prior to welding to prevent cracking [4,5].

Typically, components are preheated to 300–350 °C before welding. In post-welding, while the joint is still hot, the part is placed in a furnace at 600 °C to homogenize the temperature throughout the volume, followed by slow cooling within the furnace to prevent HAZ cracking. This results in a material with low hardness suitable for machining.

If no further processing is required, the furnace temperature is gradually raised to the quenching temperature of 880 °C, followed by supercritical cooling in warm oil, which minimizes the risk of cracking. A subsequent tempering treatment at 560 °C with air cooling is performed immediately after hardening.

Pre- and post-heating operations, however, complicate and increase the cost of the welding process. Consequently, alternative welding conditions have been explored, where the part is vibrated during welding and cooling to reduce internal stresses, deformities, and the risk of cracking.

Vibration-assisted welding (VAW) leverages the inherent internal energy of matter, comprising kinetic and potential energies of constituent particles. In metals, this includes atomic vibrations within the crystal lattice, electron orbital movements, and spin. While internal energy is influenced by pressure, volume, and temperature, temperature exerts the most significant effect on solids. Vibrations induce pressure variations, thereby affecting internal energy.

Thermal energy input elevates the material’s temperature, increasing atomic kinetic energy and vibrational energy. Thermal expansion also increases the material’s volume, creating more space between atoms and a higher vacancy count. Additional vibrations alter internal energy, affecting both kinetic and potential components, with the extent of change depending on vibration amplitude, frequency, and material properties.

This research enhances the VAW process, building on the principles of Vibratory Stress Relief (VSR), a method widely used to reduce residual stresses in castings or welded structures. VSR involves subjecting objects with high internal stresses to vibrations with high amplitude and low frequency. These mechanical oscillations alleviate weld stresses and redistribute them, reducing crystal lattice distortions and bringing the material closer to equilibrium, thus stabilizing dimensions. Optimal results are achieved when vibrations resonate with the object [6,7].

The equipment utilized includes an electromagnetic vibrator or a motor with an unbalanced mass, rigidly attached to the stressed object. An accelerometer controls the VSR system, maintaining resonance frequency or another frequency that ensures large-amplitude oscillations.

Over time, various VAW methods have been developed to reduce manufacturing costs by eliminating the need for VSR or PWHT. VAW not only reduces HAZ stresses but also affects the solidification process, refines crystalline grains in the weld bead, prevents Widmanstätten structure formation, and enhances mechanical properties and weld bead aesthetics.

Recent VAW advancements are detailed in [8,9,10,11]. Mechanical oscillations in the welding process are typically generated by vibrating the part at a resonant frequency using a vibrator from the VSR process at frequencies below 150 Hz [12,13,14,15,16,17]. Higher-resonance frequency vibrations are achieved with electromagnetic or electrodynamic vibrators [18,19,20,21]. Our research utilized a vibrating table capable of creating quasi-resonance conditions across a wide frequency range up to 9500 Hz, allowing for controlled acceleration directionality.

Mechanical oscillations can also be induced locally in the molten metal area through various methods, such as the Cold Metal Transfer (CMT) welding process [22], high-frequency oscillations with piezoelectric transducers, voltage pulses over DC welding current, and vibration of filler rods in TIG or wires in MIG/MAG welding [23,24,25,26,27]. While these techniques minimally impact the base metal, they significantly improve the weld bead’s microstructure and mechanical characteristics.

This study emphasizes the importance of the resultant acceleration direction—along the weld cord, transverse to it, and vertically—and contributes to a deeper understanding of VAW.

## 2. Experimental Section

### 2.1. Research Aims and Objectives

The experiments aimed to achieve welded joins without pre- and post-heating of low-weldability steels by applying a VAW procedure. The main objective was to detect optimal welding–vibrating regimes to avoid welds cracking and to achieve a safe and reliable joint.

### 2.2. Materials

Our research employed 42CrMo4 steel, a low-alloy steel with a medium carbon content. This specific material was chosen due to its exceptional combination of strength and wear resistance. 42CrMo4 boasts a range of impressive properties that make it ideal for applications experiencing demanding loads and harsh operating conditions. The high strength of 42CrMo4 allows it to withstand significant static and dynamic loads. This makes it a perfect choice for car engines, gearboxes, and heavy machinery components. Additionally, its superior fatigue resistance enables it to endure repeated stress cycles without failure, a crucial characteristic for components like crankshafts and gears. Furthermore, 42CrMo4 exhibits excellent low-temperature toughness. This ensures good impact resistance even in extreme cold environments, making it valuable for equipment operating under harsh conditions. These combined properties contribute to the widespread use of 42CrMo4 steel in the automotive industry, particularly for engine and gearbox components. Its suitability extends beyond that, as 42CrMo4 can serve as a base material for components requiring enhanced wear resistance. This is because the steel can readily receive a hard layer deposited through welding, creating a perfect combination of strength and wear resistance. This makes 42CrMo4 an optimal choice for demanding applications in heavy mining equipment, excavators, leveling equipment, and stone transport vehicles.

**Figure 1:**
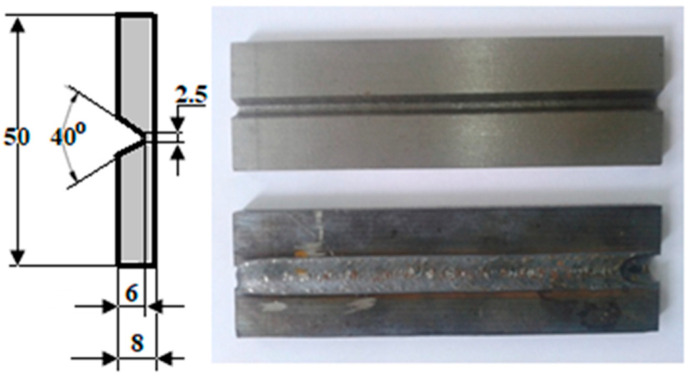
Shape and dimensions of test samples.

The material used in this work was delivered in a normalized state. Round bars with a diameter of 250 mm (D = 250 mm) were used as the starting material for the specimens. Discs with a thickness of 10 mm (t = 10 mm) were cut from these bars using a band saw. To minimize microstructural changes and maintain the normalized state, a cutting method with minimal heat input, such as a band saw, was chosen. Discs obtained from the bars were then milled to create the final samples shown in Figure 1. The chemical composition and hardness of the sample material are presented in Table 1.

Butt welding of 10 mm thick material is usually performed in two passes. The root layer was welded with low linear energy, and the second layer at much higher energy. The samples have a 2 mm extra material bottom at the base of the V joint. This base was preserved and allowed to replace the root layer. For the joint filling layer, the automat synergic mode of the welding equipment was imposed, corresponding to the welding of unalloyed construction steel with 0.2%C.

### 2.3. Samples

From the 42CrMo4 steel, 14 samples were prepared with the shape and dimensions shown in Figure 1.

### 2.4. The Equipment Used to Apply the VAW Process

**Figure 2:**
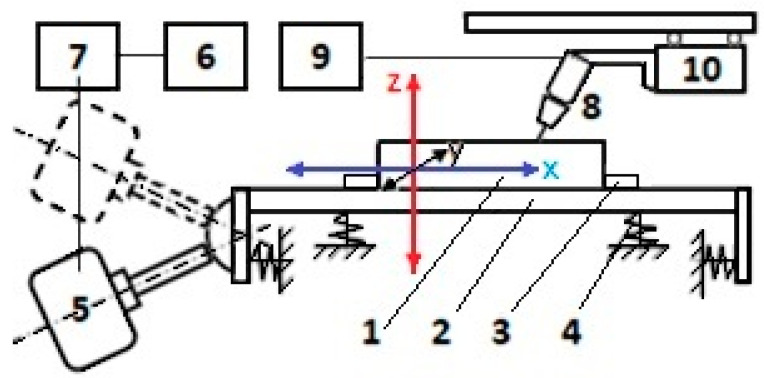
Vibrating welding table scheme.

Figure 2 shows the scheme of the vibrating table on which the specimens were welded under the conditions of applying the VAW process. The patent for this equipment was obtained as follows: Vibrating table for welding, RO127504B1 [28].

Part (sample) 1, subjected to the welding operation by the VAW process, was placed on vibrating platform 2. In order not to move under the action of the vibration, the part was held in position by stop 3. The elastic system 4 allowed platform oscillations in the x, y, and z axes, with different accelerations in each direction, depending on the position of the electrodynamic exciter 5. The low-frequency generator 6 emitted a sinusoidal or stepped electrical signal which was amplified by the power amplifier 7 and transmitted to the electrodynamic exciter. This generated mechanical oscillations with a maximum frequency of 18 kHz and adjustable amplitude. Welding was performed with the welding torch 8, powered by the MIG/MAG source 9. The torch’s constant-speed movement was ensured using the welding tractor 10.

Unidirectional oscillations generated on different frequencies by the electrodynamic exciter propagate in the vibrating platform in the form of longitudinal waves, transverse waves, or planar waves. For this reason, the vibrations recorded on the platform surface in different areas, in the center, at the edge, or the corners, have different values and orientations. The unloaded platform vibrates in a certain way, and under load, the vibration conditions change, these being influenced by the weight of the vibrated part as well as by the area where it is placed.

The oscillation frequency and the elastic wave propagation mode influence both the platform oscillations and those of the piece placed on it. By changing the part position or the exciter concerning the vibrating platform, and by adjusting springs in the elastic support system of the platform, it is possible to achieve the desired acceleration in each direction and in any area, a_x_-a_y_-a_z_. Platform and part vibrations were measured with an accelerometer coupled to a vibrometer.

The increase in the frequency and the acceleration in the welding axis direction determined the formation of a weld with small scales and consequently reduced unevenness. Higher accelerations in the transverse direction (a_y_) reduced the elevation, leading to the weld flattening. Vibration must be carried out in such a way as to produce a sufficiently large vertical acceleration (a_z_) but not exceed a certain limit, as in such cases gaseous inclusions—pores—accumulate in the weld.

**Figure 3:**
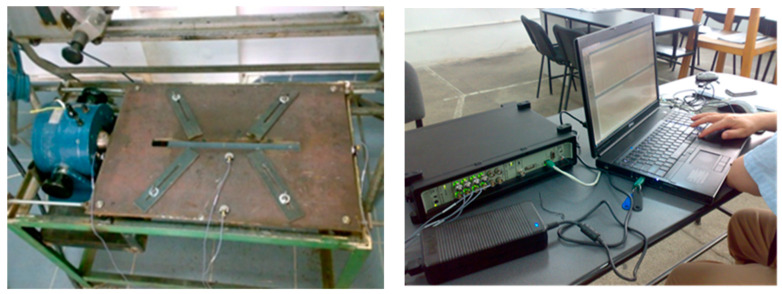
Dynamic signal analyzer pulse system—Type 3560 C-E01.

The oscillations analysis of the vibrating table, in different positions of the electrodynamic exciter concerning the vibrating platform, was performed with the Type 3560 C-E01 Bruel & Kjaer signal analyzer system, presented in Figure 3.

**Figure 4:**
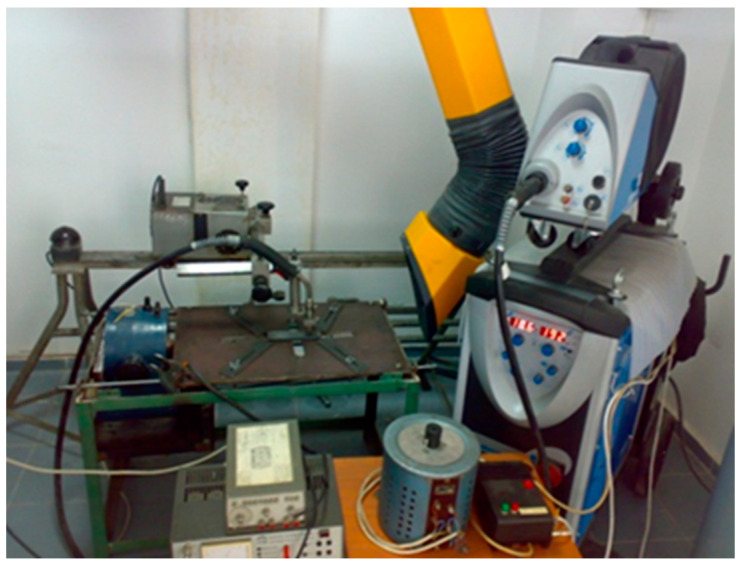
Equipment used for vibration-assisted welding.

**Figure 5:**
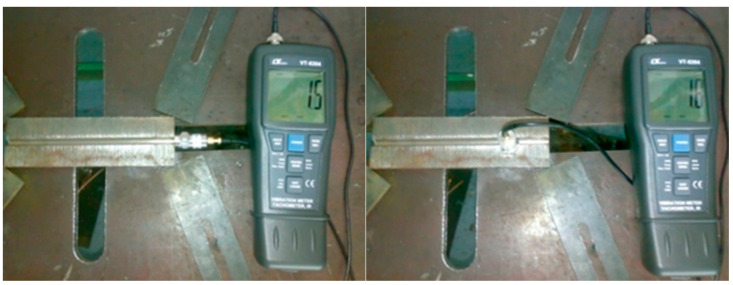
VAW acceleration measurement in the weld direction a_x_, and in the perpendicular direction a_z_.

In addition to the vibrating table and its related devices (frequency generator and power amplifier), the assembly carried out included (Figure 4) the following:

- MIG/MAG source—SAF RO Digipuls Inverter 320, capable of performing synergistic welding;
- ETAB welding tractor, controlled with direct current, for the constant and adjustable movement of the MIG/MAG welding torch;
- PCE-VT204 vibrometer (Figure 5), which ensures the measurement of oscillations with the accuracy of acceleration 0.1 m/s^2^; velocity 0.1 mm/s; and displacement 0.001 mm, used to measure the accelerations of each specimen in the x- and z-directions, the acceleration in the y-direction being negligible.

### 2.5. Physical Characteristics That Characterize the Specimen Vibration

An elastic medium is constituted of interacting particles. A disturbance propagates through this medium from close to close, with finite speed. If under a force action, one of the particles begins to oscillate, the disturbance is transmitted to the others, and the motion propagates in the form of a mechanical (elastic) harmonic wave. The phenomenon is described by the variation in time and space of the physical quantities that characterize the microvolume ΔV of the respective environment: displacement, speed, acceleration, pressure, and density [29].

The displacement (ξ) manifested in a certain direction is described by the equation:

$$\xi \left(x,t\right)=Acos(\omega t-kx)$$

wherein A is the oscillation amplitude. The other physical characteristics disturbed by the elastic wave are:

- Oscillation speed: $u\left(x,t\right)=-\omega A\sin(\omega t-kx)$
- Oscillation acceleration: $a\left(x,t\right)=-\omega^{2}Acos\left(\omega t-kx\right)$
- Elastic deformation: $\varepsilon \left(x,t\right)=kA\;sin\left(\omega t-kx\right)$
- Pressure variation: $p\left(x,t\right)=\sigma =\varepsilon E=EkA\;\sin(\omega t-kx)$
- Density variation: ρ(x,t)=ρkA sin⁡(ωt−kx)

where k is the spring constant (1/m).

The propagation of the elastic wave in a given environment involves a continuous energy transfer from source to environment. The energy thus transferred is found in the environment as energy to shake the environment particles around their equilibrium positions and as potential deformation energy. Consider a microvolume ΔV, which has the mass Δm = ρ·ΔV, in the medium in which a unidirectional elastic wave propagates.

The kinetic energy of the considered element is:

$$\Delta E_{c}=\frac{\Delta m\cdot u^{2}}{2}=\frac{\rho ∆V\cdot \omega^{2}A^{2}}{2}{sin}^{2}(\omega t-kx)$$

Considering that ${∆E}_{p}=\frac{k^{*}\cdot {\left(∆l\right)}^{2}}{2}$, $k^{*}=\frac{S\cdot E}{l_{0}}$, $∆l=\varepsilon \cdot l_{0}$, the potential energy equation is:

$$∆E_{p}=\frac{S\cdot E\cdot l_{0}\cdot \varepsilon^{2}}{2}=\frac{∆V\cdot E\cdot k^{2}\cdot A^{2}}{2}{sin}^{2}(\omega t-kx)$$

where: S—cross section surface; E—elasticity modulus.

but the wave propagation speed is: $v_{l}=\sqrt{\frac{E}{\rho }}$, → $E=\rho \cdot v^{2}$, and $k=\frac{\omega }{v}$,
which then results in: $∆E_{p}=\frac{\rho \cdot ∆V\cdot \omega^{2}\cdot A^{2}}{2}{sin}^{2}(\omega t-kx)$

It was found that the two energies were equal: ΔEc = ΔEp; and the total energy of the considered element was: ΔW = ΔEc + ΔEp = 2ΔEc.

The energy density was w = ΔW/ΔV

$$w\left(x,t\right)=\rho \cdot \omega^{2}\cdot A^{2}\cdot {sin}^{2}\left(\omega t-kx\right)=\rho \cdot u^{2}$$

The average value of the energy density, ⟨w⟩, represents the average during one oscillation period:

$$\langle w\rangle =\frac{1}{T}\int_{0}^{T}wdt=\frac{\rho \cdot \omega^{2}{\cdot A}^{2}}{2}\int_{0}^{T}\frac{1-cos[2(\omega t-kx)]}{2}dt=\frac{\rho \cdot \omega^{2}{\cdot A}^{2}}{2}$$

The wave intensity is the average throughout the energy flow density:

$$I=\left\langle J_{en}\right\rangle =\left\langle w\right\rangle \cdot v=\frac{v\cdot \rho \cdot \omega^{2}A^{2}}{2}=\frac{Z\omega^{2}A^{2}}{2}$$

where Z represents the acoustic impedance. Acoustic impedance (Z) is a physical medium property which describes how much resistance a sound beam encounters. Specific acoustic impedance is defined as:

$$Z=\rho \cdot v[\frac{10\;kg}{m^{2}\cdot s}=rayl]$$

Conclusion: The wave intensity depends on both the source characteristics (A, ω) and those of the environment (ρ = v·Z). The pressure equation’s results for the maximum pressure are cxv as follows:

$$I=\frac{p_{max}^{2}}{2\rho v}=\frac{p_{max}^{2}}{2Z}$$

Different steel brands have physical and acoustic characteristics that vary within certain limits, so that the propagation of elastic waves manifests itself differently [30]:

- Longitudinal wave speed: v_l_ = 5660–5900 m/s;
- Transverse wave speed: v_t_ = 2790–3240 m/s;
- Acoustic impedance: (45.4–46.7) × 10^6^ kg/m^2^·s;
- Density: ρ = 7710 … 8030 kg/m^3^.

In this paper, the main physical quantities that characterize the mechanical oscillations to which the specimens were subjected during welding were calculated to evaluate the influences of vibrations on the welded joints. The following values were used for the calculation: Z = ρ·v (ρ ≈ 7800 kg/m^3^, v ≈ 5700 m/s) → Z ≈ 44.5 × 10^6^ kg/m^2^·s.

Accelerations were measured with a vibrometer:

- In the welding bead direction—a_x_;
- Vertical to the vibrating table—a_z_;

The following were calculated:

- Effective acceleration →$a_{ef}=\sqrt{a_{x}^{2}+a_{z}^{2}}[\frac{m}{s^{2}}]$
- Maximum pressure →$p_{max}=Z\cdot \frac{a_{ef}}{\omega }[\frac{N}{m^{2}}];$
- Wave intensity →$I_{ac}=\frac{1}{2}Z\frac{a_{ef}^{2}}{\omega^{2}}\left[\frac{N}{m\cdot s}\right];$
- Energy density →$\left\langle w\right\rangle =\frac{1}{2}\rho \omega^{2}A^{2}=\frac{1}{2}\rho \frac{a_{ef}^{2}}{\omega^{2}}=\frac{Z{\cdot a}_{ef}^{2}}{2\cdot v\cdot \omega^{2}}[\frac{J}{m^{3}}]$

### 2.6. Specimens’ Vibration and Welding Regime

Before starting the VAW research program, several tests and verifications were performed to choose the welding regime, following which the synergistic welding regime was chosen for a non-alloy steel with 0.2%C, with thickness h = 5.4 mm, which in the conditions of welding without vibrations showed welds with a good aspect. The used filler material was SG2—1.2 mm, mild steel welding wire.

Preliminary welding tests were performed, and it was established that for V-joint welding, with the dimensions shown in Figure 1, the optimal welding regime was d_e_ = 1.2 mm, U_a_ = 18 V, I_s_ = 185 A, v_e_ = 4.2 m/min, v_s_ = 13.8 cm/min, Q = 15 L/min, and El = 11.6 kJ/cm, applied using the welding device SAF RO Digipuls 320. This welding regime was applied to all the samples presented in this study. The vibration conditions, frequency, and oscillation acceleration varied, but the welding parameters remained unchanged.

To vibrate the specimens, the electrodynamic exciter was fixed in a horizontal position, and the vibrating platform springs were compressed by 10 mm and 5 mm for each spring. Under these conditions, the B … I specimens were vibrated in quasi-resonance mode at different frequencies: 550 … 9600 Hz. The vibration regime for the welded specimens is presented in Table 2.

Sample A was welded employing preheating at 300 °C and post-heating for 30 min at 600 °C, followed by a gradual cooling within the furnace. Although this sample exhibited a less desirable surface appearance, it was characterized by a minimal hardness in the heat-affected zone (HAZ) and the greatest bending angle before fracture, indicative of the maximum ductility achievable in this welded steel.

Sample S, on the other hand, was welded without any vibration assistance and devoid of pre- and post-heating treatments. Despite its visually appealing finish, the weld quality was inferior, marked by elevated hardness in the HAZ, pronounced brittleness, and a reduced angle of fracture in the bend test, denoting lower quality.

Samples S and A serve as the polar benchmarks, with all other vibrated samples falling within this spectrum. This paper delves into the detailed outcomes derived from four representative samples, providing a comprehensive analysis of the effects of vibration-assisted welding on weld quality.

## 3. Results

### 3.1. Aspect

**Figure 6:**
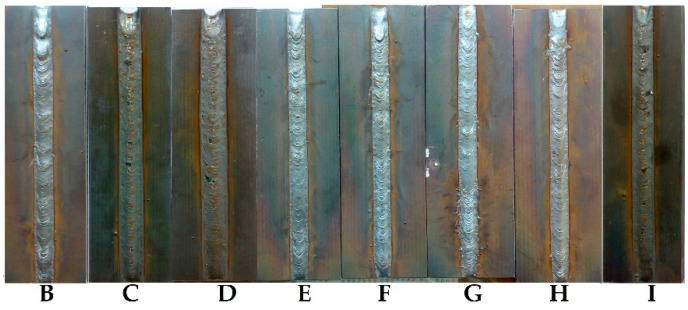
Aspect of VAW samples.

**Figure 7:**
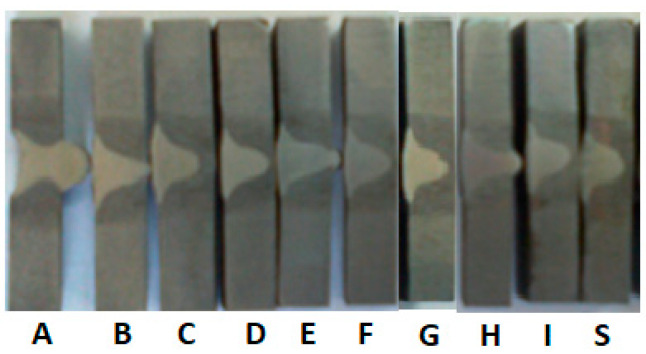
Cross-section of the welded samples.

The vibrated welds aspect is shown in Figure 6. It is observed that in samples H and I, vibrated with frequencies above 6000 Hz, the scales of the weld bead are closer. Samples A, B, and S are not present here, but these can be seen in Figure 7, as they were photographed after the bending test.

Figure 7 shows the cross-sectional appearance of the welded samples. The vibration influence on weld penetration can be observed. As vertical acceleration increases, joint root penetration increases. Sample A presents a surface with the greatest height variations and pierced root.

**Figure 8:**
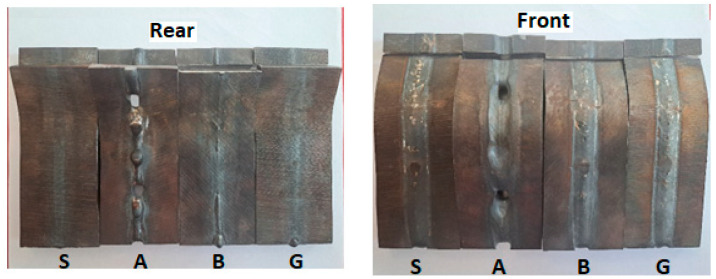
Image of reference samples (the coding of the samples corresponds to that given in Table 2).

The vibration influence analysis on welded joints was based on an in-depth comparative study made on four samples that present reference characteristics. Their image is shown in Figure 8 after the bending test was performed.

Sample S, which was not vibrated or preheated, has a suitable appearance, but in the bending test, a high fragility was recorded. The aspect of sample B, at the limit, is unacceptable because the weld root is very close to being pierced at the vibration regime: 550 Hz, a_ef_ = 50 m/s^2^. Sample G was vibrated with a less energetic acceleration (a_ef_ =20 m/s^2^), resulting in a corresponding aspect.

### 3.2. The Bending Tests

The bending test is a simple qualitative analysis to evaluate both ductility and material strength. In general, this experiment tested either rigid or medium-flexible samples made of various materials: metal, plastic, wood, or ceramic. A bending test produces tensile stresses on the convex sample side and compressive stresses on the concave side.

**Figure 9:**
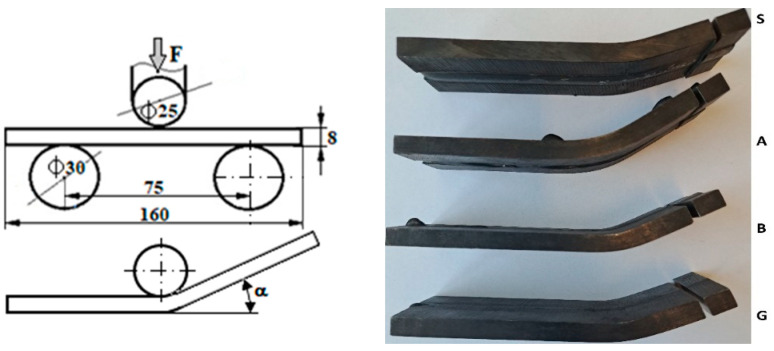
Bending test (the letters next to the representative samples correspond to those given in Table 2).

The three-point bending test (Figure 9) is a classic mechanics experiment for the material’s elastic Young modulus determination. A beam with length L rests on two rollers and is subjected to a concentrated load F in its center. A sample’s behavior in this test depends on its microstructure, texture, material tensions, and internal imperfections [31].

In the bending test, the specimen is placed in the center of the testing device. The support rollers are arranged parallel to each other at a certain distance (support width). The pressing mandrel, which moves slowly and at a constant speed, loads the sample with an increasing force until a crack of about 3 mm appears. The maximum load during the bending test represents the breaking force. The test was performed on a universal testing machine: Traktionsprüfgerät FU 10000e Rauenstein. The obtained results are presented in Table 3.

This test highlights both the fragility of the base material and of the deposited material resulting from the welding process. When tested, the welded cord was subjected to tensile stresses. The only sample that cracked in HAZ was the S sample, which was welded without preheating and without vibration. In all other specimens, cracking in the weld occurred. This finding indicates that the vibration greatly reduces the stresses in the HAZ and the fracture occurred in the bead welded with SG2. Sample S has a minimum deformation capacity, sample A has the best deformability, and the vibrated samples B and G have intermediate values but are close to those obtained for the pre- and post-heated sample A.

### 3.3. Macrostructure

**Figure 10:**
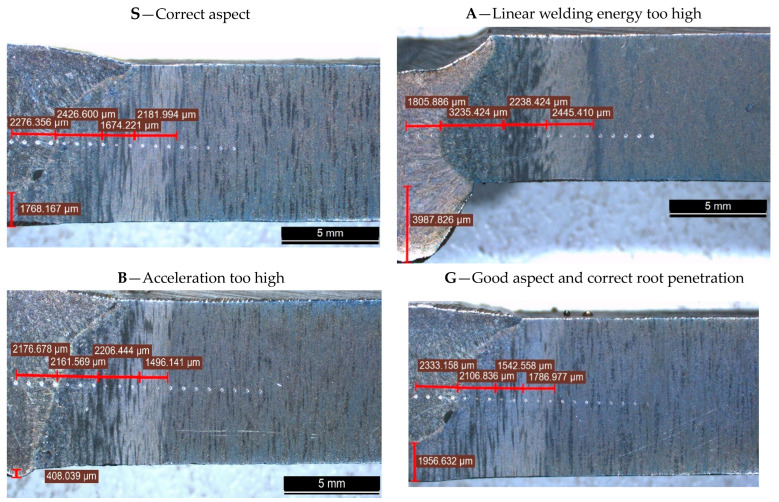
Macrostructure of the welds and Vickers prints for micro-hardness measurement.

After performing the bending test, a metallographic proof was detached from one sample end for macro- and microscopic analysis, as well as for the micro-hardness measurement per section around the weld bead. The samples were taken at a distance of 10 mm from the sample end of the welding seam by transverse cutting with a water jet (with the abrasive jet cutting machine MAXIEM1530). For the macro- and microscopic study, the samples were sanded, polished, and developed with NITAL 6 reagent for 5 s and photographs and measurements were taken of areas with different appearances in the HAZ. For this purpose, a LEICA S9D macroscope equipped with LAS V4.10 software was used. Figure 10 presents the macrostructure and remarks about the welded samples.

In the preheated but not vibrated sample A, the joint root is pierced, and molten material has leaked out. The conclusion is drawn that in the case of preheating, the linear welding energy must be greatly reduced to achieve a quality joint. This welding regime was preserved only for a comparative highlight of the VAW effects at constant welding parameters. Sample A, even if it did not have a good aspect, was considered a reference sample that presented the lowest internal stresses and hardness in HAZ.

Sample S has a proper macro appearance, the joint root is well welded, and it is confirmed that the welding regime is suitable for welding without vibration and preheating.

Four distinct zones can be observed in the HAZ with different sizes and aspects. These are indicated in Table 4.

**Figure 11:**
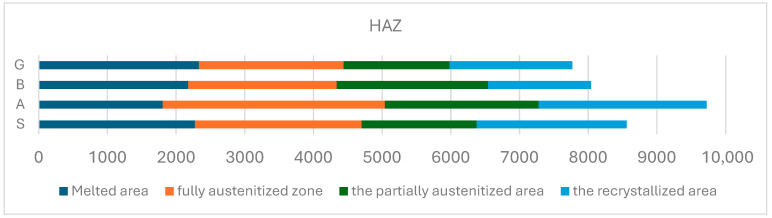
The graph of the dimensions of the specific areas in the HAZ.

From Figure 11, the low size of the HAZ in the vibrated samples and a greater depth of heat propagation can be observed. Therefore, it can be stated that mechanical vibrations influence the solidification and crystallization and direct the heat in the sense of increasing the penetration.

### 3.4. Micro-Hardness

For the hardness measurement, the DuraVision 250 G5 hardness tester was used, which provides fully automatic measurements. The chosen measurement type was HV5, with an indentation step of 0.7 mm. The measurement was made at 3 mm below the sample surface.

Table 5 shows all the hardness measurements provided by the durometer.

**Figure 12:**
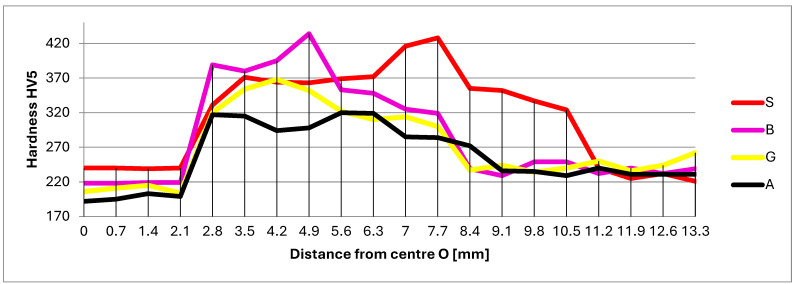
Hardness variations in the welded area.

Figure 12 shows the four reference samples, with the hardnesses Vickers visualization. The hardness measurement starts from the center of the filler material (point 1), continues with the fusion line (p. 4, 5), goes through the HAZ (p. 7 … 9), and continues in the base material to point 20.

As a first observation, the bar (D = 250 mm) used to obtain samples has an accentuated texture oriented in the lamination direction. This was highlighted after etching. Thus, the segregation traces were oriented perpendicular to the welded samples. The HV harness variation is presented in Figure 12.

In the filler materials, sample S (240 HV) presented the highest HVmedium, and the preheated sample A (≈197 HV) has the lowest hardness. The vibrated samples were placed between these two values (210–220 HV). It is also found that the HAZ width was the maximum in sample S. The preheated sample A and the vibrated sample B and G have a comparable width of HAZ, but was about 3 mm smaller (11.2–8.4 mm). Sample G shows a small hardness variation (320–368 HV). Sample B has a peak hardness of 434 HV. This can be explained by the presence of segregation that generated micro-zones with different chemical compositions and implicitly with other hardnesses.

At the vibrated samples, further than 9 mm from the middle of the weld, the base material does not show changes in microstructure and hardness.

### 3.5. Microstructure

**Figure 13:**
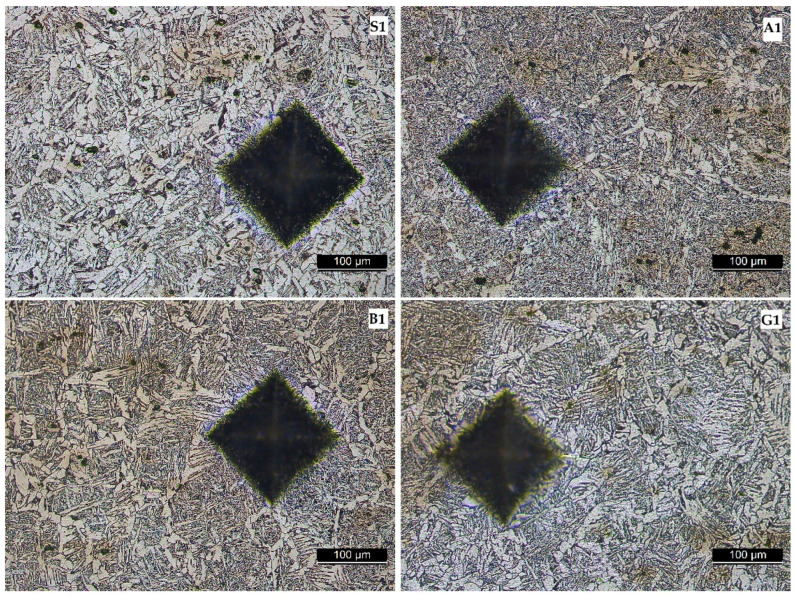
Microstructure of deposited material in the welded seam.

**Figure 14:**
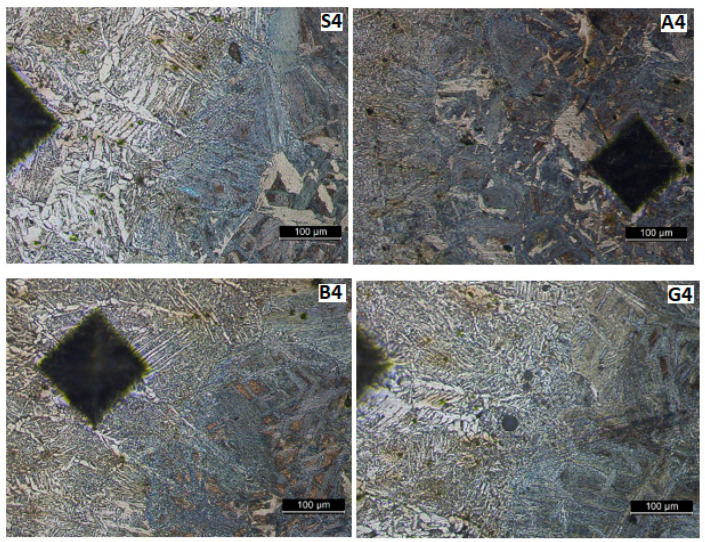
Microstructure in the contact zone weld bead—base material.

**Figure 15:**
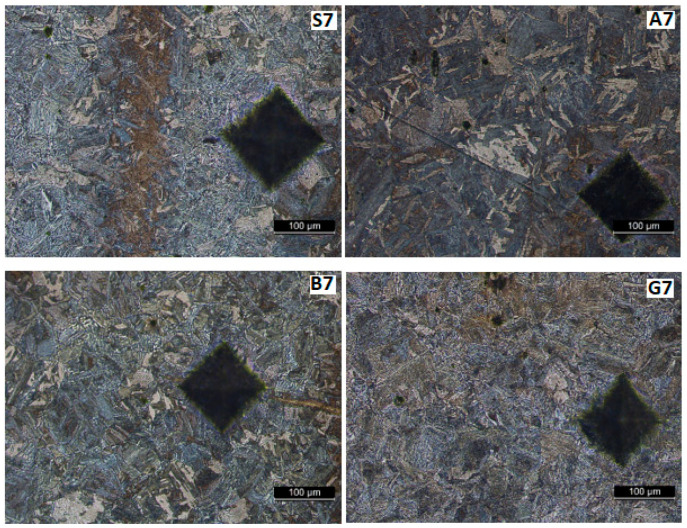
Microstructure in HAZ at 5 mm from the weld center.

**Figure 16:**
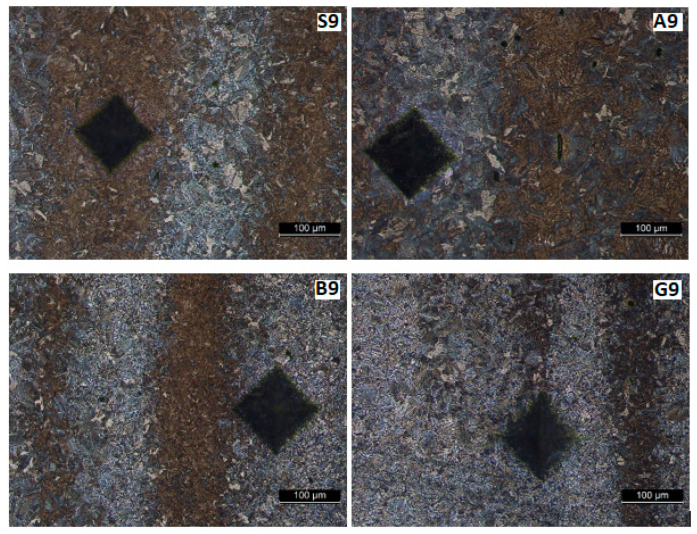
Microstructure in HAZ at 6.5 mm from the weld center.

Figure 13, Figure 14, Figure 15 and Figure 16 show the microstructures of the welded samples. The symbol indicates the sample (S, A, B, G) and the micrograph location with the Vickers indentation number.

S1 = sample S, point 1 (middle of the weld);
S4 = sample S, point 4 (fusion line);
S7 and S9 = sample S, point 7 or 9 (in HAZ);
S20 = Sample S, point 20 (base material).

The same applies for samples A, B, and G.

While the focus of this research is on the base material, the filler material, SG2, also warrants some discussion. Its chemical composition is primarily 0.06–0.13% carbon (C), 0.7–1.00% silicon (Si), and 1.30–1.60% manganese (Mn) [32]. Due to the very low carbon content, the SG2 material does not form extensive martensitic zones, which are hard and brittle. Therefore, the white, needle-like structures observed in Figure 13 are composed of ferrite, a softer and more ductile phase. In the welded seam, a Widmanstatten structure is identified [33]. This structure typically forms under conditions of high heat input and rapid cooling. In our vibrated samples, however, the distribution and refinement of the phases within the Widmanstatten structure appear more pronounced. This suggests that vibrations may reduce the amount and negative effects of the Widmanstatten structure.

The presence of a dilution zone (a gradual interpenetration zone) between the deposited material and the base material indicates that the fusion line is conforming (Figure 14) with respect to the welding regime applied. Consequently, the microstructure and properties change relatively uniformly from the deposited material (on the left side of the micrographs—Figure 14) towards the base metal, which has a higher carbon content and greater hardness (located on the right side of the images in Figure 14 and with a darker appearance).

Figure 14 and Figure 15 show the HAZ microstructure. No important differences are observed between the vibrated and non-vibrated samples. It is found that in areas with segregations, Vickers indentations are smaller in these zones, while hardness is bigger.

**Figure 17:**
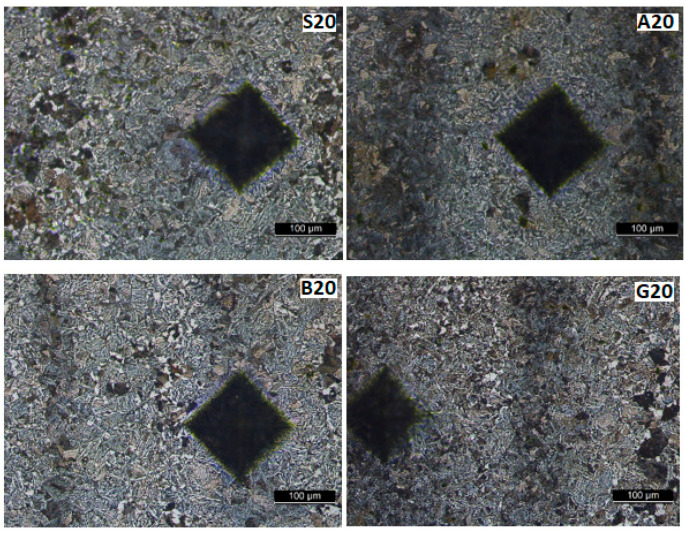
Microstructure in base material at 14 mm from the weld center.

The macrostructure of the base material shown in Figure 17 is consistent across all samples. Darker vertical bands are visible on these microstructures (Figure 15, Figure 16 and Figure 17) after development. These bands can arise from two sources: residual internal stresses resulting from plastic deformation during rolling or segregation of alloying elements (Mn, C …) elongated during rolling in the semi-finished product. Since the certificate of conformity accompanying the semi-finished product indicates that it was delivered in a normalized state (and traces of plastic deformation should no longer be visible), this appearance should be attributed to the segregation of alloying elements, leading to the formation of rows in which pearlite grains predominate. In this zone, the hardness is higher, and the Vickers press mark is smaller. Regardless, this aspect cannot be attributed to the influence of vibrations, as it originates from the production of the semi-finished product.

## 4. Discussion

Figure 6 and Figure 7 reveal a noticeable alteration in the appearance of VAW-welded beads, both on the surface and in their cross-sections. Since only the vibration regime was modified, it is evident that these dimensional changes in the weld beads are directly attributed to the vibration. Notably, samples B, E, and H (Figure 7), vibrated during welding with accelerations of 50 m/s^2^, 49 m/s^2^, and 48 m/s^2^, respectively, exhibit a significantly enhanced weld penetration.

The bending test results (Table 3) indicate that samples B, G, and H, vibrated with accelerations exceeding 20 m/s^2^, demonstrate clear changes in their mechanical properties. These samples exhibit reduced brittleness and improved ductility, with their bending angles approaching that of the pre- and post-heated welded sample.

Analyzing the macrostructure (Figure 10 and Figure 11), a noticeable change in the dimensions of specific zones within the heat-affected zone (HAZ) is evident. Specifically, vibrated samples B and G display a significantly smaller HAZ.

Hardness variations must be interpreted separately for the filler material and base material due to their differing chemical compositions. Comparing the HVmed. hardness values in the filler materials of representative samples (Table 5), it can be concluded that vibrated samples have lower HVmed. values (HVmed. B = 219, HVmed. G = 209) compared to the non-vibrated sample (HVmed. S = 240) and approach the hardness values of the pre- and post-heated sample (HVmed. A = 197).

A similar trend is observed for the average hardness values in the HAZ of the base material. Vibrated samples exhibit lower average hardness values (HVmed. B = 319 and HVmed. G = 296) compared to the non-vibrated sample (HVmed. S = 356), but these values are slightly higher than those of the base material in the pre- and post-heated sample (HVmed. A = 280).

Therefore, it can be observed that the application of the VAW process leads to a decrease in the overall hardness of both the filler material and the thermally affected base material. The decrease in hardness is inversely proportional to the excitation accelerations (a_ef_) applied to the samples during welding. This means that sample B, excited with a_ef_ = 50 m/s^2^, has a higher average hardness (in both zones) compared to sample G, excited during welding with a_ef_ = 20 m/s^2^.

Despite its higher hardness, sample B exhibits better ductility (30°) than sample G (28°) (Bending test, Table 3). This phenomenon can be explained by the refinement of the microstructure with increasing acceleration. This grain refinement justifies the observed increase in hardness alongside improved ductility, further supported by the variation in deforming force in the bending test (F_B_ = 6020 daN and F_G_ = 4875 daN). Grain refinement leads to an increase in both yield strength (R_p_) and ultimate tensile strength (R_m_).

The microstructure analysis reveals a pearlitic structure with segregation banding. In the HAZ, due to the welding heat input and cooling rate, phase transformations occur, leading to the formation of structures with diverse morphologies. The weld contains typical types of ferrite for unalloyed weld metal. The morphology of ferrite depends on the observation location and the cooling rate of the joint after welding.

## 5. Conclusions

The VAW process utilizes a vibrating table to introduce controlled vibrations during MIG/MAG welding. By adjusting the exciter position and spring tension on the vibrating platform, the system can achieve various frequencies (50 Hz–10 kHz) and accelerations in multiple directions (x, y, and z). This study found that vibrations between 550 and 9500 Hz significantly affect the appearance and properties of the weld. Notably, the back-and-forth shaking (oscillation acceleration) along the welding direction (axis) plays a crucial role in shaping the final weld. Additionally, the direction of vibration has a distinct effect: sideways shaking (transverse acceleration) influences the seam width, while up-and-down shaking (vertical acceleration) determines how deeply the weld penetrates the base material. However, using excessive vibration (over 50 m/s^2^) can lead to defects like molten metal expulsion or penetration through the bottom of the weld.

The research identified a minimum acceptable vibration acceleration of 20 m/s^2^, as demonstrated by sample G. This level achieved a satisfactory cracking angle in the bending test. Weld quality was assessed by comparing the average hardness of the filler material and the zone in the base material that was heated by welding (the heat-affected zone from HAZ). The results showed that the sample welded without vibration (S) had the highest HAZ hardness and lowest ductility, indicating a more brittle structure. Conversely, the preheated sample (A) displayed the lowest hardness and highest ductility, signifying a more favorable and ductile structure. The vibrated samples presented properties in between these two extremes, with sample B, excited at 50 m/s^2^, exhibiting characteristics closest to the preheated sample.

A significant advantage of VAW lies in its energy efficiency. The vibration generation system requires minimal power (100 W) compared to the high energy consumption of pre- or post-heating furnaces. Additionally, preheating adds complexity to the welding process, creates a less desirable working environment for welders, and ultimately diminishes productivity.

## Figures and Tables

**Table 1:** Chemical composition and hardness of sample material (42CrMo4).

C	Mn	Si	P	S	Cr	Mo	Cu	HV
0.42	0.78	0.38	0.031	0.033	1.11	0.18	0.22	220–240

**Table 2:** The vibration mode of samples in automatic welding with constant linear energy.

Parameters	Established			Measured		Calculated			
Sample	Exciter		f	a_z_	a_x_	a_ef_	p_max_	I_ac_	〈w〉
	V	W	Hz	m/s^2^	m/s^2^	m/s^2^	daN/cm^2^	kN/ms	J/m^3^
S	without vibration, without pre- and post-heating								
A	without vibration, T_pre_ = 300 °C(≈T_Ms_) + T_post_ = 600 °C, 30 min								
B	16	80	550	40	30	50.000	643.275	4.654	0.816
C	13	65	750	30	20	36.056	340.173	1.301	0.228
D	7	35	1250	23	30	37.802	213.991	0.515	0.090
E	18	90	3000	43	25	49.739	117.319	0.155	0.027
F	6	30	4500	20	11	22.825	35.892	0.014	0.003
G	25	125	5100	20	1	20.025	27.784	0.009	0.002
H	20	100	6500	35	33	48.104	52.367	0.031	0.005
I	25	125	9600	11	0.7	11.022	8.124	0.001	0.000

**Table 3:** Bending test results.

Sample	S	A	B	C	D	E	F	G	H	I
Force [daN]	4960	4500	6020	5325	4220	4965	4500	4875	4310	4720
Angle [^o^]	18	39	30	18	17	25	20	28	27	21

**Table 4:** The dimensions of the specific areas in the HAZ.

Area	Type of Heating	Sample	S	A	B	G
1	melted area (center)		2276	1806	2177	2333
2	fully austenitized zone (dark grey)		2427	3235	2162	2106
3	the partially austenitized area (striped grey)		1674	2238	2206	1543
4	the recrystallized area (light gray)		2182	2445	1496	1787
5	Total HAZ		8559	9724	8041	7769

**Table 5:** Hardness values per section.

Indentation	Distance from the Center	Hardness HV5 on Sample			
Point	[mm]	S	A	B	G
p. 1	0	240	192	218	206
p. 2	0.7	240	195	218	211
p. 3	1.4	239	203	219	215
p. 4	2.1	240	199	219	204
HVmed. in filler materials		240	197	219	209
p. 5	2.8	331	317	389	320
p. 6	3.5	371	315	380	354
p. 7	4.2	364	294	395	368
p. 8	4.9	363	298	434	352
p. 9	5.6	369	320	353	322
p. 10	6.3	372	319	348	310
p. 11	7	416	285	325	314
p. 12	7.7	428	284	319	300
p. 13	8.4	355	272	239	237
p. 14	9.1	352	236	229	244
p. 15	9.8	337	235	249	234
p. 16	10.5	324	229	249	240
p. 17	11.2	240	240	232	250
HVmed. in HAZ		356	280	319	296
p. 18	11.9	225	231	240	236
p. 19	12.6	232	231	232	244
p. 20	13.3	221	231	239	262

## Data Availability

The raw data can be available on request from the corresponding author.
